# Metastatic adenocarcinoma involving the right ventricle and pulmonary artery leading to right heart failure: case report: Adenocarcinoma metastático envolvendo o ventrículo direito e artéria pulmonar levando a insuficiência cardíaca direita: relato de caso

**DOI:** 10.1590/1516-3180.2016.0351280117

**Published:** 2017-05-29

**Authors:** Turgut Karabag, Caner Arslan, Turab Yakısan, Aziz Vatan, Duygu Sak

**Affiliations:** I MD. Associate Professor, Department of Cardiology, Istanbul Education and Research Hospital, Istanbul, Turkey.; II MD. Associate Professor, Department of Cardiovascular Surgery, Cerrahpasa Medical School, Istanbul University, Istanbul, Turkey.; III MD. Resident Physician, Department of Cardiology, Istanbul Mehmet Akif Ersoy Education and Research Hospital, Istanbul, Turkey.; IV MD. Resident Physician, Department of Emergency Medicine, Istanbul Education and Research Hospital, Istanbul, Turkey.; V MD. Resident Physician, Department of Internal Medicine, Istanbul Education and Research Hospital, Istanbul, Turkey.

**Keywords:** Neoplasm metastasis, Adenocarcinoma, Echocardiography, Heart failure, Pulmonary artery

## Abstract

**CONTEXT:**

Obstruction of the right ventricular outflow tract due to metastatic disease is rare. Clinical recognition of cardiac metastatic tumors is rare and continues to present a diagnostic and therapeutic challenge.

**CASE REPORT:**

We present the case of a patient who had severe respiratory insufficiency and whose clinical examinations revealed a giant tumor mass extending from the right ventricle to the pulmonary artery. We discuss the diagnostic and therapeutic options.

**CONCLUSION:**

In patients presenting with acute right heart failure, right ventricular masses should be kept in mind. Transthoracic echocardiography appears to be the most easily available, noninvasive, cost-effective and useful technique in making the differential diagnosis.

## INTRODUCTION

Tumors involving the heart are more commonly metastatic than primary, and the prognosis for metastatic tumors in the heart is extremely poor. Involvement of the right heart is more common than that of the left heart and the clinical course is usually silent in most patients.^1^ This report presents the case of a 67-year-old male patient with no previous diseases, in whom a metastatic right cardiac tumor invading the right ventricular outflow tract and the pulmonary artery was detected. We also discuss the diagnostic and therapeutic techniques.

## CASE REPORT

A 67-year-old male patient was admitted to the emergency department with a 15-day history of progressive fatigue, shortness of breath and respiratory insufficiency. The patient stated that he had not had any complaints until 15 days before admission, and no previous diseases had been documented. He reported having progressive shortness of breath, which first appeared during exercise 15 days earlier and had then even become apparent at rest. The patient reported having made intermittent use of paracetamol for headache for years. He had a history of smoking and intermittent alcohol use.

On admission, the patient’s general condition was poor, with severe shortness of breath. He was orthopneic and was using accessory muscles while breathing. His blood pressure was 80/65 mmHg, and his pulse was 128/minute. His breathing sounds were rough but no rales or rhonchi were heard. Heart sounds were tachycardic, and there was a 2/6 systolic murmur heard in the tricuspid area. He had 1+ edema of both feet. A chest x-ray showed increased vascularization, a dilated pulmonary artery and a cardiothoracic index of > 1. An electrocardiogram revealed an incomplete right bundle branch block and a negative T wave in the V1-3 leads.

Laboratory tests conducted on venous blood sample showed that blood glucose was 124 mg/dl (normal range 74-100); urea, 90.2 mg/dl (normal range 0-50); creatinine, 1.6 mg/dl (normal range 0-1.2); sodium, 134 mg/dl (normal range 132-146); and potassium, 5.2 mg/dl (normal range 3.5-5.5). Liver function tests revealed elevated values: aspartate transaminase, 3418 U/l (normal range 0-50); alanine transaminase, 1204 U/l (normal range 0-50); alkaline phosphatase, 276 U/l (normal range 30-120); and lipase, 92 U/l (normal range 0-67). Total bilirubin and direct bilirubin levels were slightly elevated: 8.4 g/dl (normal range 6.6-8.3) and 0.24 mg/dl (normal range 0-0.2), respectively. The hemogram was unremarkable except for leukocytosis. The prothrombin time was 21.2 seconds and was slightly lengthened (normal range 10.4-14.6 seconds). Cardiac markers were also slightly elevated: troponin I, 0.45 ng/ml (normal range 0-0.001); and creatinine kinase-myocardial band, 6.4 ng/ml (normal range 0.6-6.3). An arterial blood gas test revealed pH of 7.49, sO_2_ of 87.9%, pO_2_ of 57.2 mmHg and pCO_2_ of 22.5 mmHg.

**Figure 1.**
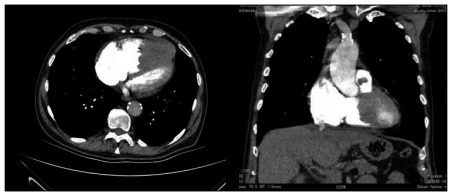
Contrast computed tomography pulmonary angiogram showing a dilated right ventricle and a filling defect, both in the right ventricle and in the pulmonary artery.

In the light of these data, the patient received an initial diagnosis of acute toxic hepatitis, taking into consideration the history of pulmonary embolism and recent use of medication. A complete abdominal ultrasound examination revealed a slightly enlarged liver. A contrast computed tomography pulmonary angiogram was performed and did not show any thrombus in the major arteries or their branches. On the other hand, a filling defect from the right ventricle to the pulmonary artery, which was interpreted to represent a thrombus, was observed (Figure 1).

**Figure 2.**
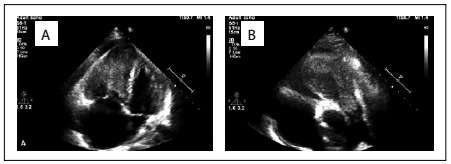
Echocardiogram showing the mass inside the right ventricle, which extended to the pulmonary artery and invaded the pulmonary valve, in apical four-chamber view (A) and parasternal short-axis view (B).

Therefore, transthoracic echocardiography was performed, revealing a considerably enlarged right ventricle and a flattened interventricular septum, which had shifted towards the left ventricle. A 6 cm x 5 cm mass, consistent with thrombus echogenicity, was detected inside the right ventricle. It extended to the pulmonary artery, with invasion of the pulmonary valve, giving rise to to-and-fro motion in each systole (Figure 2).

Right ventricular systolic function was considerably decreased (tricuspid annular plane systolic excursion, TAPSE: 1.6 cm). Left ventricular systolic function was slightly decreased. Severe tricuspid and mitral insufficiency was present. Pulmonary artery systolic pressure was elevated (60 mmHg).

**Figure 3.**
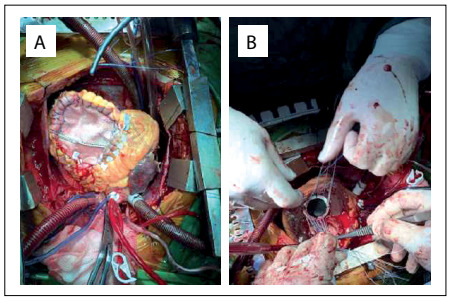
Resection of tumor from the heart, with closure of the ventricular septal defect using a patch (A) and replacement of the tricuspid valve (B).

Thus, the patient was referred for consultation in the department of cardiovascular surgery and was immediately scheduled for emergency surgery. During surgery, it was found that the mass was a tumor. The tumor had invaded the anterior wall of the right ventricle, interventricular septum and right ventricular outflow tract. The tumor was removed from the interventricular septum and the anterior wall of the right ventricle. The resulting ventricular septal defect was closed with a patch (Figure 3a) and the tricuspid valve was replaced (Figure 3b). Intraoperative transesophageal echocardiography revealed only moderate mitral insufficiency, and therefore mitral valve replacement was not considered.

**Figure 4.**
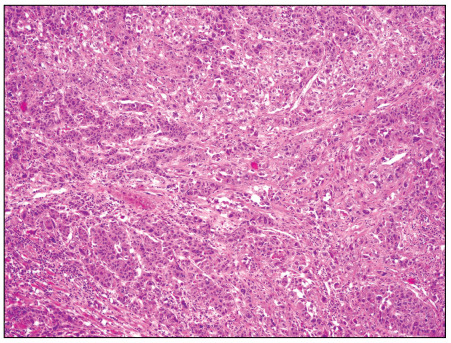
Adenocarcinoma infiltration to the myocardium. Tumor cells form coarse solid trabecular structures (100 x; hematoxylin and eosin).

The pathology report showed that the mass was a poorly differentiated metastatic adenocarcinoma, which had probably originated from the lungs. It exhibited partial neuroendocrine differentiation (Figure 4).

The patient’s postoperative hemodynamic condition did not improve, despite inotropic support. He developed cardiopulmonary arrest on postoperative day 3 and died, despite resuscitation attempts.

## DISCUSSION

Tumors that are metastatic to the heart are rare. Cardiac involvement at autopsy has been described in 6% to 20% of patients with malignant neoplasms.^2^ The lungs have been reported to be the most common primary origin of metastatic neoplasms, followed by nonsolid neoplasms such as lymphoma or leukemia and tumors of the liver and colon, respectively.^3^ The epicardium is the most commonly involved site, followed by the myocardium and the endocardium.^4^

The systematized results from searching the literature through the main databases are presented in Table 1. The data in the literature show that diagnosing and treating tumors located in the right ventricular outflow tract is extremely challenging. The symptoms usually depend on the size and location of the tumor. The most common symptoms include shortness of breath, syncope and cyanosis.^5^ Some patients may present with nonspecific symptoms and tumors are usually detected incidentally. Inability to measure blood pressure with the patient in the seated position is likely to indicate occlusion of the pulmonary artery by the tumor.^5^ These tumors may reach huge sizes, thus resulting in occlusion of the right ventricular outflow tract and right ventricular volume overload and dilation, which is likely to result in severe right ventricular failure.^6^ In addition, neoplasms may cause fatal clinical consequences including arrhythmia, acute heart failure and sudden death. Tumors may disintegrate into fragments, thus embolizing the pulmonary vascular bed. Accordingly, patients may present with clinical signs and symptoms of pulmonary embolism. Emergency surgery should be considered upon detection of right ventricular outflow tract obstruction.^4^ Early diagnosis enables timely surgical treatment, thus increasing survival. In addition, the data in the literature indicate that postoperative adjuvant chemotherapy may be useful in patients with adenocarcinomas of gastrointestinal origin that are metastatic to the heart.^7^^,^^8^

**Table 1.** Systematic search of the literature performed in April 2016

Database	Search strategies	Found	Used
MEDLINE (via PubMed)	(metastatic adenocarcinoma) and (heart)	1,388	4
LILACS (via Bireme)	(metastatic adenocarcinoma) and (heart) and (pulmonary)	0	0
Cochrane Library	(metastatic adenocarcinoma) and (heart) and (failure)	30	0

In the case presented here, the patient presented with a 15-day history of progressive shortness of breath. He showed clear signs and symptoms of right ventricular failure. A diagnosis of pulmonary embolism was initially considered, but firstly contrast computed tomography and then transthoracic echocardiography revealed an intracardiac tumor. Upon detection of obliteration of the right ventricular outflow tract and invasion of the pulmonary valve by the tumor, the patient was sent for emergency surgery. Despite successful excision of the mass, the patient’s postoperative hemodynamic condition did not improve, even though inotropic support was provided, and he subsequently died.

Although the tumor was diagnosed pathologically, the origin of the tumor could not be identified. Pathologically, the tumor was likely to have originated from the lungs, gastrointestinal tract or pancreaticobiliary system. However, this could not be confirmed because the patient’s family did not give permission for an autopsy.

## CONCLUSION

Right ventricular metastatic tumors are relatively rarer than other types. In patients presenting with shortness of breath with rapid progression and right heart failure, right ventricular obstruction should be kept in mind, along with other possible diagnoses. Transthoracic echocardiography appears to be the most easily available, noninvasive, cost-effective and useful technique in making the differential diagnosis.
